# Genetic Control and High Temperature Effects on Starch Biosynthesis and Grain Quality in Rice

**DOI:** 10.3389/fpls.2021.757997

**Published:** 2021-12-17

**Authors:** Hua Zhang, Heng Xu, Yingying Jiang, Heng Zhang, Shiyu Wang, Fulin Wang, Ying Zhu

**Affiliations:** ^1^State Key Laboratory for Managing Biotic and Chemical Treats to the Quality and Safety of Agro-products, Institute of Virology and Biotechnology, Zhejiang Academy of Agricultural Science, Hangzhou, China; ^2^College of Chemistry and Life Sciences, Zhejiang Normal University, Jinhua, China

**Keywords:** starch biosynthesis, regulatory mechanism, rice eating quality, amylose content, high temperature

## Abstract

Grain quality is one of the key targets to be improved for rice breeders and covers cooking, eating, nutritional, appearance, milling, and sensory properties. Cooking and eating quality are mostly of concern to consumers and mainly determined by starch structure and composition. Although many starch synthesis enzymes have been identified and starch synthesis system has been established for a long time, novel functions of some starch synthesis genes have continually been found, and many important regulatory factors for seed development and grain quality control have recently been identified. Here, we summarize the progress in this field as comprehensively as possible and hopefully reveal some underlying molecular mechanisms controlling eating quality in rice. The regulatory network of amylose content (AC) determination is emphasized, as AC is the most important index for rice eating quality (REQ). Moreover, the regulatory mechanism of REQ, especially AC influenced by high temperature which is concerned as a most harmful environmental factor during grain filling is highlighted in this review.

## Introduction

Rice is one of the most important staple foods, feeding more than half of the population in the world. Developing varieties with high quality is a major aim for rice breeders (James et al., 2003; Jeon et al., 2010). Starch accounts for more than 80% of the storage material in the rice endosperm and is composed of 10–30% amylose (AM) and 70–90% amylopectin (AP). AM mainly contains hundreds of glucose units with linear linkages, while AP contains thousands of glucose units and is highly branched through the α-1,6-glycosidic bond based on amylose (Takeda et al., 1990). Rice eating quality (REQ) is mainly assessed by three main physicochemical characteristics: the amylose content (AC), gel consistency (GC), and gelatinization temperature (GT; Juliano, 1985). The AC is the most important index for REQ, as it is the key determinant of the firmness and sticky nature of cooked rice (Tian et al., 2009; Tao et al., 2019). GC and GT are additional parameters representing the textural features of rice starch with the same AC (Cagampang et al., 1973; Gao et al., 2011; Zhang et al., 2020b). In recent years, certain novel functions of some starch synthesis genes have been revealed, and many genes involved in the regulation of seed development have been isolated. To obtain a comprehensive understanding of starch synthesis in rice, this review summarizes previous studies and hopefully uncovers some important regulatory mechanisms of seed development and quality control. The molecular regulation of rice quality, especially the AC, will be highlighted in this review.

## Genetic Basis of Amylose Content in Rice

The genetic control of rice AC is relatively complex. Genetic studies using different populations, such as doubled haploid (DH), recombinant inbred lines (RILs), BCmFn, and chromosome segment substitution lines (CSSLs), have been performed (Huang et al., 2000; Lanceras et al., 2000; Li et al., 2003, 2011; Septiningsih et al., 2003; Fan et al., 2005; Guo et al., 2007; Zheng et al., 2008; Liu et al., 2011; Zhang et al., 2020a), and a series of quantitative trait loci (QTLs) and/or genes for AC have been identified in the rice genome in the past few decades (Table 1; He et al., 1999; Tan et al., 1999; Bao et al., 2002; Wang et al., 2007a; Pandey et al., 2012; Fasahat et al., 2014; Takemoto-Kuno et al., 2015; Lau et al., 2016). It is now well established that *Wx* on chromosome 6 is the major locus for rice AC and has been detected in almost all studies (Wang et al., 1995; Hirano and Sano, 1998). The *Wx* gene encodes granule-bound starch synthesis I (GBSSI), which is the key enzyme for amylose synthesis in rice (Cai et al., 1998; Huang et al., 2020b). Two alleles, *Wx^a^* and *Wx^b^*, were widely distributed in *indica* and *japonica* cultivars, respectively (Cai et al., 1998). Subsequently, more allelic variations of *Wx*, such as *Wx^op^*, *Wx^mq^*, *Wx^in^*, and *wx*, were isolated (Sato et al., 2002; Mikami et al., 2008; Liu et al., 2009; Zhang et al., 2019b, 2020c; Zhou et al., 2020). Variation in *Wx* can explain most of the significant alterations of rice AC in nature. QTLs on chromosome 3 have also been explored for AC modification in many different populations, such as *Oryza sativa indica* × *Oryza sativa japonica* (Lanceras et al., 2000; Zhang et al., 2019a), *Oryza sativa indica* (Swarna) × *Oryza nivara* (Swamy et al., 2012), and *Oryza sativa indica* (Caiapo) × *Oryza glaberrima* (Aluko et al., 2004). Interestingly, all the loci from *indica* varieties have a positive effect on AC. Their similar genetic effects and close genetic location indicate that they might represent the same locus. We named this locus *qSAC3* in our previous study (Zhang et al., 2019a). Compared with the *Wx* allele, *qSAC3* has a minor effect on rice AC. In the *japonica* background, introducing the *indica* allele of *qSAC3* could mildly increase AC, and this locus was used for marker-assisted selection to improve the cooking and appearance quality in soft rice with low AC (Zhang et al., 2019a). In addition to *Wx* and *qSAC3*, two QTLs, *qAC8-1* and *qAC8-2*, responsible for AC regulation, were also identified in multiple studies (Wan et al., 2004; Wang et al., 2007b; Li et al., 2011; Liu et al., 2011). Moreover, many other QTLs for AC were detected and distributed on all rice chromosomes, although most of them showed unstable effects across different populations or different environments. Environmental factors, such as temperature, light, and soil, were found to affect rice quality obviously, while temperature shows the greatest impact on rice AC. CSSLs with same *Wx* allele planted in different seasons and different locations was used to assay the AC variation (D-value) under different environments, most CSSLs showed varied D-value with their parents lines. Such results suggested that most QTLs responsible for AC determination are not stable under varying environments, and we deduced that these loci might be involved in genetic-environment interactions of AC control. Thus, AC is genetically controlled by the major locus *Wx* and several minor loci, such as *qSAC3*, *qAC8-1*, and *qAC8-2*, which could stably affect AC under multiple conditions. Fine mapping and characterization of the candidate genes of *qSAC3*, *qAC8-1*, and *qAC8-2* will help us to understand their relationship with *Wx* and establish the exact genetic basis of AC control.

**Table 1:** Reported QTLs for amylose content of rice.

Parents/population type	Locus	Chr.	Marker/Location	Reference
ZYQ8(*indica*) × JX17(*japonica*)/DH	*qAC-5*	5	RG573~C624	He et al., 1999
	*Wx*	6	*Waxy*	
KDML105(*indica*) × CT9993(*japonica*) /RILs	–	3	C515~RM81	Lanceras et al., 2000
	–	4	GA2-7~G177A	
	*Wx*	6	*Waxy*~RM204	
	–	7	OSR22~RM10	
KDML105(*indica*) × CT9994(*japonica*) /RILs	–	3	GA1-2~R2170	Huang et al., 2000
	–	4	G177A~GA 2-7	
	–	4	C16-3~T11-5	
	*Wx*	6	R1962~RZ588	
	–	6	RG64~T11-1	
	–	9	G103~R1687	
	–	11	RG1094A~GA4	
IR64 (*indica*) × Azucena (*japonica*)/DH	–	7	RG375~RG477	Bao et al., 2002
Kasalath (*indica*) × Nipponbare (*japonica*)/BILs	*qAC-3*	3	R1927~R3226	Li et al., 2003
	*qAC-4*	4	C1100~R1783	
	*qAC-5*	5	C624~C128	
	*qAC-6, Wx*	6	R2869~R1962	
IR64 (*indica*) × *Oryza rufipogon*/BC_2_F_2_	*Wx*	6	RM170	Septiningsih et al., 2003
IR24 (*indica*) × Asominori (*japonica*)/CSSLs	*qAC-8*	8	G1149~R727	Wan et al., 2004
	*qAC-9a*	9	XNpb36~XNpb103	
	*qAC-9b*	9	C609~C506	
	*qAC-12*	12	XNpb189-2~XNpb24-2	
IR24 (*indica*) × Asominori (*japonica*)/CSSLs	*qAC-8*	8	G1149	Wang et al., 2007b
	*qAC-9*	9	X36	
IR24 (*indica*) × Asominori (*japonica*)/CSSLs	*qAC-1a*	1	XNpb113	Liu et al., 2011
	*qAC-1b*	1	R1982	
	*qAC-2*	2	XNpb67	
	*qAC-6*	6	C688	
	*qAC-8*	8	G1149	
	*qAC-9a*	9	XNpb36	
	*qAC-9b*	9	XNpb13	
	*qAC-11*	11	C1350	
IR26 (*indica*) × Asominori (*japonica*)/CSSLs	*qAC-8-1*	8	RM7356~RM7556	Li et al., 2011
	*qAC-8-2*	8	RM23510~RM23579	
Caiapo (*indica*) × *Oryza glaberrima* /DH	*amy3*	3	RM7~RM251	Aluko et al., 2004
	*amy6,Wx*	6	RM190~RM253	
	*amy8*	8	RM230~RM264	
Zhenshan 97 (*indica*) × H94 (*indica*)/DH	*ac6a, Wx*	6	RM190~RM587	Fan et al., 2005
	*ac6b*	6	*C* gene~MRG5119	
	*ac11*	11	RM209~RM229	
	*ac12*	12	RM270~RM235	
Zhenshan 97 (*indica*) × Delong (*japonica*) /DH	–	2	RM183~RM573	Wang et al., 2007a
	*Wx*	6	RM586~MX21	
	–	9	RM296~RM105	
Yuefu (*japonica*) × IRAT109 (*japonica*) /DH	*QAc3*	3	RM60~C814	Guo et al., 2007
	*QAc6, Wx*	6	C1004~R1962	
	*QAc8*	8	R2676~C166	
	*QAc9*	9	R79~R2638	
	*QAc11a*	11	RM202~RM287	
	*QAc11b*	11	G181~G320	
Zhenshan 97 (*indica*) × Minghui 63 (*indica*) /RILs	*qAC-1-1*	1	R753~G359	Zheng et al., 2008
	*qAC-1-2*	1	C904~R2632	
	*qAC-4-3*	4	C56~C820	
	*qAC-6-4, Wx*	6	C952~Waxy	
Swarna (*indica*) × *Oryza nivara* /BC_2_F_2_	*ac2.1*	2	RM262~RM3515	Swamy et al., 2012
	*ac3.1*	3	RM22~RM7	
	*ac3.2*	3	RM85~RM293	
	*ac6.1, Wx*	6	RM314~RM3	
9311 (*indica*) × Nipponbare (*japonica*)/CSSLs	*qHAC4*	4	13.4~15.9 Mb	Zhang et al., 2014
	*qHAC8a*	8	0.7~1 Mb	
	*qHAC8b*	8	8.7~21.2 Mb	
	*qHAC10*	10	19.8–20.5 Mb	
9311 (*indica*) × Nipponbare (*japonica*)/CSSLs	*qSAC3*	3	6.9~8.2 Mb	Zhang et al., 2019b
Kuiku162 (*japonica*) × Itadaki (*japonica*) /BC_1_F_4_	*qAC2*	2	RM1211	Takemoto-Kuno et al., 2015
9311 (*indica*) × PA64s (*indica*)/CSSLs	–	3	SNP3-191~SNP3-273	Zhang et al., 2020a
	*Wx*	6	SNP6-1~SNP6-11	

## Role of Starch Biosynthesis Enzymes in Endosperm Development of Rice

Many key enzymes, such as ADP glucose pyrophosphorylase (AGPase), granule-bound starch synthase (GBSS), soluble starch synthase (SS), starch branching enzyme (SBE), and starch debranching enzyme (DBE), are involved in starch synthesis in rice seeds. Most of the enzymes that have isozymes and isoforms preferentially expressed in endosperm are responsible for starch synthesis in rice seeds, such as GBSS1 (also called Wx), SS1, SS2a (also called SSIIa/SSII-3), SS3a (also called SSIIIa/SSSIII-1), SBE1 (also called BE1), and SBE2 (also called BE2b/SBEII). Previous studies proposed that AM and AP were synthesized by different enzymes in rice. AM is mainly synthesized by GBSS1, while AP is synergistically regulated by multiple enzymes, such as SSs, SBEs, and DBEs (James et al., 2003; Jeon et al., 2010). However, recent studies have improved our understanding of the functions of SSs, which might participate in the synthesis of both AM and AP, thus affecting the rice AC.

### SSs Is Essential for the AP Synthesis of Rice

It is well established that SS1, SS2a, and SS3a are responsible for AP chain elongation, while SBE1 and SBE2 control the formation of branched structures in AP (Pandey et al., 2012). The chain length distribution or degrees of polymerization (DP) in AP shows very important effects on rice quality and starch physicochemical properties (Buléon et al., 1998). The activity of rice SS1 is higher than that of SS2a and SS3a in rice endosperm. SS1 preferentially synthesizes short chains of DP 6–12. In the *ss1* mutant, chains of DP 8–12 are decreased, whereas DP 6–7 chains are increased, which indicates that SS1 elongates DP 6–7 chains to DP 8–12 chains of AP (Fujita et al., 2006; Li et al., 2018). SS3a is another important enzyme for AP synthesis, and the activity of SS3a is higher than that of SS2a but lower than that of SS1. *SS3a* is mainly responsible for the generation of long chains (DP ≥ 30) in AP (Fujita et al., 2007). The *ss3a* mutant showed significantly reduced long chains of AP and abnormal starch granule morphology, which results in a floury endosperm (Ryoo et al., 2007). This result suggested that long chains catalyzed by SS3a are critical for maintaining normal structures of starch granules. In contrast, no obvious starch granule or morphological defects were observed in *ss1* seeds (Fujita et al., 2006). However, the *ss1/ss3a* double mutant of *japonica* rice is sterile (Fujita et al., 2011; Hanashiro et al., 2011). These data indicated that the reduction of short chains in AP might not be enough for morphological alteration of starch granules, while the simultaneous reduction of both short and long chains could affect the formation of starch granules. SS2a was proposed to mainly produce intermediate chains (DP 13–25) of AP (Umemoto et al., 2002). The activity of SS2a is significantly different between *indica* and *japonica*. *SS2a* from *japonica* might be an inactive allele showing no or very low activity *in vitro*, while the *indica* allele has relatively higher activity (Umemoto et al., 2004). *SS2a* is a key gene that mainly determines rice GT, an important physiochemical property for rice eating and cooking quality (Gao et al., 2003, 2011). Introducing the *indica SS2a* allele into *japonica* rice could convert the structure of AP from the S-type (mainly in *japonica* cultivars) to the L-type (mostly in *indica* cultivars) and increase GT significantly as well (Nakamura et al., 2005).

### SSs Might Play an Important Role in AM Synthesis of Rice

It was generally believed that SSs (SS1, SS2a, and SS3a) were only involved in AP synthesis. Recent studies noted that these SSs might also affect AM synthesis. The short chains of AP produced by SSs could supply substrates for the synthesis of AM (Zhu et al., 2020). SS1 is a dominant enzyme for AP synthesis, especially for the short chain of DP 6–12, as its activity accounts for approximately 70% of the total SS activity (Fujita et al., 2006). Thus, deficiency of *SS1* would cause a great reduction in AP. However, the appearance of seeds and starch granules remained normal, and the AC remained unchanged in the *ss1* mutant (Fujita et al., 2006). Moreover, *sbe2* mutant seeds present a higher AC than wild-type seeds (Butardo et al., 2011), and knockdown of *SS1* in *sbe2* results in AC compensation (Abe et al., 2014). We deduced that the increase of AC in total starch was due to greatly impaired AP synthesis in *sbe2*, whereas defects in AM biosynthesis subtly balanced the ratio of AM to AP and returned the AC to WT level in the double mutant generated from leaky mutant of *SS1*crossed with *sbe2* mutant. These results strongly suggested that *SS1* plays important roles not only in AP synthesis but also in AM synthesis, and the short chains of AP (DP 6–12), which are mainly produced by SS1, might be important substrates for AM synthesis.

*SS2a* might be another *SS* gene involved in AM synthesis. Introducing the high activity allele *SS2a^Ind^* (*indica* allele of *SS2a*) into rice plants could raise the AC whereas the effects are ecotype dependent (Yang et al., 2018; You et al., 2020). In the *Wx^a^* background, *SS2a^Ind^* could increase AC dramatically (Tian et al., 2009), while in the *Wx^b^* background, *SS2a^Ind^* has a minor effect on AC (Zhang et al., 2020b). The total activity of the GBSSI protein generated by *Wx^a^* is higher than that generated by *Wx^b^* (Wang et al., 1995). Therefore, we deduced that the intermediate chains of AP (DP 13–25) produced by *SS2a* might be substrates for AM synthesis, and *Wx^a^* may use these substrates with higher efficiency than *Wx^b^*. It will be very interesting to investigate the AC alteration in which *SS2a^Ind^* is introduced into the genetic background with weaker *Wx* alleles, such as *Wx^mq^* and *Wx^hp^*. *SS3a* might not be involved in the synthesis of AM. Loss of function of *SS3a* caused a significant reduction in long chains (DP > 30) in AP and no obvious alteration in AM synthesis (Fujita et al., 2007), although the relative ratio of AM to AP was increased and the AC was increased in the *ss3a* mutant. These results indicated that long chains of AP produced by SS3a might not be used as substrates in AM synthesis. Thus, we proposed that short and intermediate chains (DP < 25) of AP might be important substrates for AM synthesis and both *SS1*and *SS2a* play important roles in this process. More evidence for this conception should be collected in the future by using other technologies, such as radio isotope tracer.

### Function of SBEs in Starch Synthesis of Rice

SBE1 and SBE2 show different enzyme activities and biological functions in starch synthesis. SBE1 presents higher activity than SBE2 in rice endosperm. SBE2 has a high affinity for AP, while SBE1 is involved in branch addition in both AP and AM (Nakamura et al., 2010). Although no significant morphological defects were found in *sbe1* seeds, both intermediate chains of DP 12–21 and long chains of DP ≥ 37 were reduced which resulted in a GT decrease in *sbe1* seeds (Satoh et al., 2003). *SBE2* seems to play a more important role in AP synthesis than *SBE1* (Zhu et al., 2012; Nakata et al., 2018; Baysal et al., 2020). Short chains (DP < 17) were decreased greatly, and opacity or chalkiness appearance occurred in *sbe2* (*ae*, amylose extender) seeds (Nishi et al., 2001; Butardo et al., 2011). This indicated that *SBE2*, similar to *SS1*, is very important for short-chain synthesis. Interestingly, in the *ss1/sbe2* (*ss1/ae*) double mutant, similar to *ss1/ss3a*, normal starch granules could not be formed, and very few seeds could be produced (Abe et al., 2014). These results suggested that AP synthesis and the chain length distribution are very important for starch granule formation, which eventually affects rice yield and quality.

## Other Essential Genes Regulate Seed Development and Grain Quality of Rice

### Key Factors and Regulatory Network in AM Synthesis and REQ Control Related With Core Gene *Wx/GBSSI*

**Figure 1:**
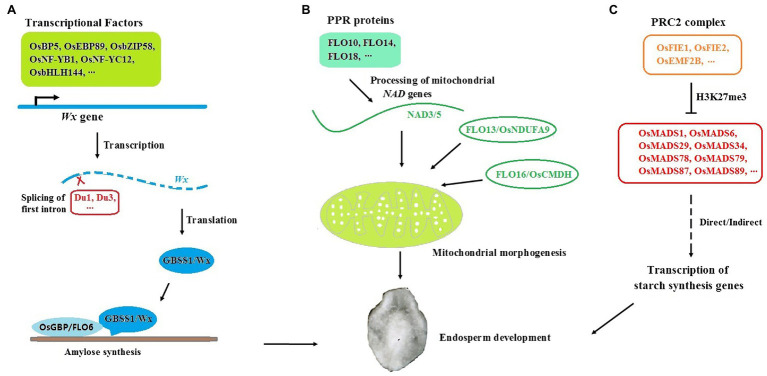
Regulation of amylose synthesis and endosperm development in rice seeds. **(A)** *Wx/GBSSI* is finely controlled at multiple levels. OsBP5, OsEBP89, OsbZIP58, OsNF-YB1, OsNF-YC12, and OsbHLH144 are transcriptional factors which could bind to the promoter of *Wx* gene and regulate its expression. Du1 and Du3 are responsible for alternative splicing of *Wx*. OsGBP and FLO6 might help the GBSS/Wx protein localized to starch in amylose synthesis. **(B)** FLO proteins (FLO10, FLO14, FLO18, FLO13, and FLO16 et al.) are involved in mitochondrial morphogenesis and endosperm development through NADH pathway. **(C)** The PRC2-MADS pathway for early seed development in rice. OsFIE1, OsFIE2, and OsEMF2B are important components of PRC2 complex. *OsMADS1*, *OsMADS6*, *OsMADS29*, *OsMADS34*, *OsMADS78*, *OsMADS79*, *OsMADS87*, and *OsMADS89* are involved in the process of PRC2-mediated early endosperm development.

As we mentioned above, amylose synthesis is mainly controlled by the *Wx* gene, and many allelic *Wxs*, such as *Wx^a^*, *Wx^b^*, *Wx^in^*, *Wx^mq^*, *Wx^lv^*, and *wx*, explain a major AC variation in rice germplasm. With the development of biotechnology, more novel *Wx* alleles were generated and many important rice materials with different AC were produced by genetic modification or gene editing recently, and most of them occurred at coding and promoter region of *Wx* gene (Liu et al., 2014; Zeng et al., 2020; Huang et al., 2020a; Xu et al., 2021). Moreover, the *Wx* gene can be finely regulated at the transcriptional, post-transcriptional, and translational levels, and the factors involved in these processes are also important for AC modulation and rice quality control (Figure 1A).

Many transcription factors have been reported to transactivate *Wx* expression by binding to cis-elements, such as 31 bp core sequences (Ge et al., 2000) and Em boxes (Cheng et al., 2002) at the upstream of *Wx*. For example, a bHLH transcription factor, OsBP5, together with its interacting protein, OsEBP89, binds to 31 bp and synergistically regulates the transcription of *Wx* (Zhu et al., 2003). OsbZIP58, a key factor in starch synthesis, was verified to directly bind to cis-elements from both *Wx* and *SBEI* and coordinately control AP and AM biosynthesis at the transcriptional level (Wang et al., 2013). Knockout or knockdown of these genes would cause alterations in AC and rice quality.

Several *dull* genes were isolated recently, and some of them were found to regulate *Wx* expression at the post-transcriptional level. *Du1* encodes an mRNA splicing factor and participates in the splicing of the first intron in rice *Wx* (Zeng et al., 2007). *Du3* encodes a protein similar to nuclear cap binding protein subunit 2 and is involved in the nuclear export of *Wx* mRNA (Isshiki et al., 2008). The splicing efficiency of the *Wx* gene was reduced and the AC decreased significantly in both *du1* and *du3* mutants, which indicated that post-transcriptional regulation of *Wx* is very important for rice quality control. In addition to the genes that regulate *Wx* expression and GBSSI activity, an increasing number of novel factors and pathways have been revealed to influence AM synthesis. *PTST1* (Protein Targeting to Starch), which is responsible for AM synthesis in *Arabidopsis* leaves, was newly found to help the GBSS protein localize to starch (Seung et al., 2015). The CBM48 domain at the C-terminus of PTST1 is important for its binding activity to starch. Although GBSS itself has weak binding activity to starch, it could be recruited by PTST1 through the coiled-coil domain and subsequently bound to starch mediated by the CBM48 domain in PTST1 (Seung, 2020). *OsGBP*, a homolog of *PTST1* in rice, could interact with both rice *GBSS* genes, *Wx* and *GBSS2*, *in vitro*. However, only AM biosynthesis in leaves but not in endosperm was greatly impaired in the *osgbp* mutant (Wang et al., 2020). Therefore, *OsGBP* may mainly function in chloroplasts, and there might be other factors involved in GBSSI locating starch in the endosperm. *FLO6* (floury endosperm), another homolog of *PTST1* in rice, is the most likely such gene. FLO6 contains the CBM48 domain at the C-terminus and can interact with GBSSI, GBSSII, and ISA1 to help them target starch in endosperm. Total starch and AC decreased significantly, and starch granules were abnormal in the *flo6* mutant (Peng et al., 2014; Zhang et al., 2021). *FLO6* might be involved in AM synthesis in rice. The transcription factor OsNF-YB1 can bind to a G-box in the *Wx* promoter and activate its expression (Xu et al., 2016). Moreover, OsNF-YB1 can interact with several transcription factors, such as OsNF-YC12 and OsbHLH144, and form a complex to regulate starch synthesis genes, including *Wx* and *ISA1* (Bello et al., 2019). Mutants of os*nf-yb1*, os*nf-yc12*, and os*bhlh144* displayed similar phenotypes to *flo6*, such as chalky endosperm, reduced grain weight, and decreased total starch and AC. Moreover, OsNF-YC12 binds to the promoter of *FLO6* and directly regulates its expression (Xiong et al., 2019). Further study to reveal the biological function of *FLO6* and *OsGBP* and their regulatory network in GBSSI activity modification will be very meaningful for elucidating starch synthesis and quality control in rice seeds.

### *FLO* Genes Play Important Roles in Endosperm Development of Rice

In addition to *flo6*, many new *flo* mutants with floury endosperm were isolated, and most of them showed abnormal starch granules and reduced grain weight and AC (Figure 1B). Many *FLO* genes have been cloned and found to engage in different biological processes. *FLO10*, *FLO14*, and *FLO18* encode pentatricopeptide repeat (PPR) proteins involved in RNA binding and metabolism in plant mitochondria. The processing of mitochondrial *NAD* genes, such as *NAD1* and *NAD5*, was defective in *flo10*, *flo14*, or *flo18* mutants (Wu et al., 2019; Xue et al., 2019; Yu et al., 2020a). *NAD* genes encode subunits of NADH dehydrogenase that are essential for ATP production and mitochondrial development. *FLO13*, known as *OsNDUFA9*, encodes subunit mitochondrial complex I. Loss of *OsNDUFA9* changes the mitochondrial structure and greatly impairs the development of rice endosperm (Hu et al., 2018). *FLO16*, known as *OsCMDH*, encodes an NAD-dependent cytosolic malate dehydrogenase. ATP and AC were obviously reduced in the *flo16* mutant (Teng et al., 2019). These reports suggested that regulators involved in the NADH pathway are essential for both mitochondrial morphogenesis and endosperm development in rice. Moreover, *FLO2* was predicted to encode a tetratricopeptide repeat (TPR) domain-containing protein (She et al., 2010). The candidate gene responsible for *FLO4* encodes a pyruvate orthophosphate dikinase (Kang et al., 2005; Zhang et al., 2018b). *OsHsp70* is the gene responsible for the *FLO11* phenotype, and *FLO15* encodes glyoxalase I (Zhu et al., 2018; You et al., 2019). Clarifying the biological function of these *FLO* genes will be beneficial to uncover new components and pathways influencing seed development and rice quality in the future.

### PRC2-MADS Cascade Is Essential for Early Seed Development of Rice

The early development of endosperm has a great influence on the quality and yield of rice. Polycomb repressive complex 2 (PRC2), which catalyzes trimethylation of histone H3 at lysine 27 (H3K27me3), is essential for the early development of endosperm (Figure 1C; Tonosaki and Kinoshita, 2015). Fertilization-independent endosperm (FIE) is an important component of PRC2. There are two *FIE* genes, *OsFIE1* and *OsFIE2*, in the rice genome (Luo et al., 2009). Seed defect phenotypes, such as limited endosperm development, semisterile spikelets, and impaired grain size and quality, were obviously displayed in *osfie* (*osfie1* or *osfie2*) mutants (Nallamilli et al., 2013; Li et al., 2014; Huang et al., 2016; Cheng et al., 2020). Some *MADS-box* genes, which are mainly responsible for floral organ identity, seem to be involved in the process of PRC2-mediated early endosperm development. For example, *OsMADS6* plays an essential role in endosperm nutrient accumulation. In the *osmads6* mutant, starch filling was blocked, and the relative contents of protein and soluble sugar increased, which resulted in altered grain size and quality (Yu et al., 2020b). ChIP-PCR analysis revealed that H3K27 is trimethylated in vegetative tissues where *OsMADS6* is silenced (Zhang et al., 2010). Other type II *MADS* box genes, such as *OsMADS1* (Liu et al., 2018; Yu et al., 2018), *OsMADS34* (Ren et al., 2016), and *OsMADS29* (Yin and Xue, 2012; Nayar et al., 2013), also contribute to early endosperm development and might be regulated by PRC2. In rice lacking OsEMF2B, another important component of PRC2, the expression of the above *MADS-box* genes was altered (Conrad et al., 2014; Xie et al., 2015). Moreover, several type I *MADS-box* genes, such as *OsMADS78*, *OsMADS79*, *OsMADS87*, and *OsMADS89*, also played essential roles in early seed development (Paul et al., 2020). *OsMADS78* and *OsMADS79* could interact with *OsMADS87* and *OsMADS89* and form a heterodimerized complex. Transgenic seeds deficient in these type I *MADS-box* genes exhibited accelerated endosperm cellularization and altered grain quality. The expression of these *MADS* genes was negatively correlated with *OsFIE1* (Folsom et al., 2014). All these data suggested that PRC2-MADS might be an essential cascade for early seed development. As an increasing number of *MADS* genes have been found to be highly expressed in endosperm, we speculate that numerous *MADS* genes will function in PRC2-mediated early endosperm development and grain quality control in the future.

## Regulatory Mechanisms of Rice Quality at High Temperature

In addition to genetic control, environmental factors, such as temperature, light, and soil, could affect rice quality significantly as well (Li et al., 1989; Dai et al., 1998; Yamakawa et al., 2007). For instance, GT and AC of IR661 was decreased greatly under high light condition (Li et al., 1989). Production of high-quality rice was usually associated with some specific soil (Dai et al., 1998), such as black soil in Northeast of China and distinct soil infiltrated by snow water in Niigata of Japan. However, environmental temperature might have the greatest influence on rice quality. Low total starch and AC and a highly chalky appearance were often observed in *japonica* cultivars under high temperature (HT; Yamakawa et al., 2007; Zhang et al., 2016). The deterioration of rice quality under HT was thought to be mainly due to the increased grain filling rate and decreased duration of grain filling (Yamakawa et al., 2007; Zhang et al., 2018a).

### Expression and Splicing Efficiency of *Wx^b^* Is Important for Rice AC at HT

**Figure 2:**
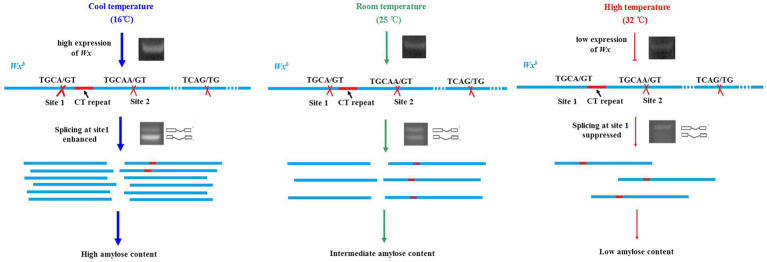
Transcriptional and post-transcriptional regulation of rice *Wx^b^* stimulated by different temperatures. Expression of *Wx^b^* is induced by cool temperature (16°C) but suppressed by high temperature (32°C). Alternative splicing at site 1 in the first intron of *Wx^b^* is suppressed by high temperature but promoted by cool temperature.

The reduction of the AC at HT is mostly caused by the downregulation of the *Wx* gene (Larkin and Park, 1999). Compared to the transcriptional inhibition of *Wx* by HT, post-transcriptional regulation induced by HT seems more important, especially in the *Wx^b^* background. A single nucleotide polymorphism (SNP, G to T) at the splicing site of the first intron in *Wx^b^* causes low splicing efficiency (Cai et al., 1998; Isshiki et al., 1998). The splicing efficiency is temperature-dependent. The splicing efficiency and mature transcripts of *Wx^b^* under cool temperature conditions (18°C) were much higher than those under optimal temperature conditions (25°C) and HT conditions (33°C). Two major mature transcripts could be generated from the *Wx^b^* allele under optimal temperature conditions. The large one is spliced after CT repeats (site 2), and the small one is spliced near the donor site of *Wx^a^* (site 1; Zhang et al., 2014). Two transcripts are generated almost equally from *Wx^b^* under optimal temperature conditions, while the large transcript represents the majority under HT, and the small transcript mainly exists at cool temperature (Larkin and Park, 1999; Zhang et al., 2014). These results suggested that the selection of donor sites in alternative splicing of *Wx^b^* is temperature-dependent. Alternative splicing at site 1 was suppressed by HT but promoted by cool temperature (Figure 2). We deduced that some important factors might control the selection of splicing sites and that the activity of these factors is sensitive to temperature.

**Figure 3:**
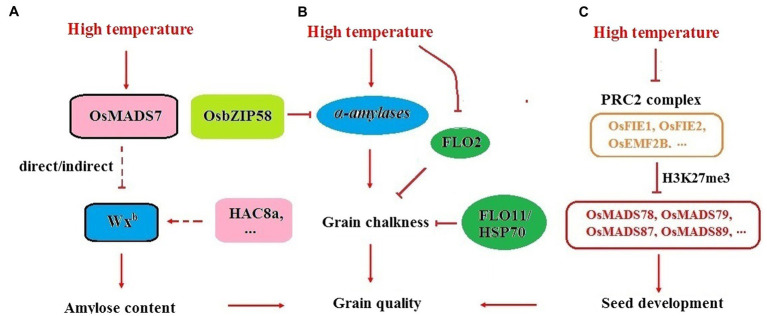
Seed development regulation and rice quality control under HT. **(A)** Increasing the efficiency of *Wx^b^* pre-mRNA processing is an important regulatory mechanism for maintaining AC stability at HT. *HAC8a* and *OsMADS7* might be important regulators involved in this pathway. **(B)** *α-amylases* is in charge of chalky appearance under HT and OsbZIP58 seems be a key gene negatively regulating *α-amylases* expression. *FLO2* and *FLO11/HSP70* are essential genes for chalkiness production under HT. **(C)** PRC2-MADS pathway might be essential for the regulation of rice seed development at HT conditions. *OsFIE1* might affect seed development through *OsMADS87*, *OsMADS89*, etc.

*Indica* rice is usually more tolerant to HT than *japonica* in terms of AC. Under the same *Wx^b^* background, the drop in AC in 9311 (*indica*) under HT was much smaller than that in Nipponbare (*japonica*). Using the CSSLs between 9311 and Nipponbare, several QTLs, *qHAC8a*, *qHAC8b*, and *qHAC4*, responsible for AC stabilization under HT were characterized. Introducing the *indica* allele of these loci into Nipponbare could enhance the splicing efficiency of *Wx^b^*, which suggested that increasing the pre-mRNA processing efficiency of the *Wx* gene might be an important regulatory mechanism for maintaining AC stability at HT (Figure 3A; Zhang et al., 2014). The results from *MADS7*-RNAi plants strongly supported this hypothesis. The floral identity gene *OsMADS7* was mildly expressed in endosperm but strongly induced by HT. Suppression of *OsMADS7* could improve the stability of rice AC under HT. Dynamic qRT-PCR revealed that both the expression level and the pre-mRNA processing efficiency of the *Wx* gene were enhanced in *OsMADS7* RNAi seeds under HT during almost the entire filling stage. *OsMADS7* might be the gene that can negatively regulate the expression or alternative splicing of the *Wx* gene under HT (Zhang et al., 2018a). Moreover, dynamic analysis revealed that grain filling rate is higher at HT than that in optimal temperature condition in both wild type ZH11 and *MADS7-RNAi* seeds. However, the difference in grain filling rate between HT and optimal temperature condition is smaller in *MADS7-RNAi* than that in ZH11, which might be another reason for relatively stable AC in *MADS7-RNAi* under HT condition (Zhang et al., 2018a).

### Other Starch Biosynthesis Enzymes Affect Rice AC at HT

In addition to *Wx*, the expression of many other starch biosynthesis genes was also changed at HT during the filling stage in rice. Overall, *SS2a* and *SS3a* were slightly downregulated, and *SS1* was induced by HT. *SBE1* changed slightly, whereas *SBE2* decreased significantly under HT (Yamakawa et al., 2007; Liao et al., 2015). Alterations in the expression of these genes are also important for rice quality control under HT. *SS3a* and *SBE2* are critical genes for AP synthesis. Significant downregulation of *SBE2* and *SS3a* indicated that AP synthesis might be impaired by HT (Zhao et al., 2020). However, AM synthesis should be much more impaired than AP synthesis, since AC was reduced greatly under HT (Lin et al., 2020). The relative abilities of AM *Vs* AP biosynthesis under HT might be lower than those under optimal temperature conditions. As mentioned above, *SS1* or *SS2a* is responsible for both AM and AP synthesis. Considering the great reduction in GBSSI activity (Zhang et al., 2014), the high expression of *SS1* at HT might be more beneficial to the synthesis of AP than that of AM, so the reduction in AC at HT should be explained in part by the high expression of *SS1*. Similar to that of *SS1*, the high activity of the *indica* allele of *SS2a* might make AC more sensitive to HT. In contrast, knocking down *SS2a* might be beneficial to improve the quality of *japonica* rice under HT because it not only reduces rice GT but also diminishes AC effects caused by HT.

### Essential Genes Regulating Seed Development and Grain Chalkiness of Rice Under HT

The PRC2-MADS pathway might also be essential for the regulation of rice endosperm development under HT conditions. Genome-wide association analysis revealed that one of the PRC2 components, *OsFIE1*, is a major locus for grain size regulation under HT conditions (Dhatt et al., 2021). The expression of *OsFIE1* in endosperm can be suppressed by heat stress. Seed development in the *osfie1* background was more sensitive to HT than that in the WT. *OsMADS87* was negatively regulated by *OsFIE1* but induced by HT. *OsMADS87* RNAi seeds were more tolerant to HT than WT seeds by using the alteration of seed size as a trait (Figure 3C; Chen et al., 2016).

Some *FLO* genes might also be responsible for seed development under HT. For example, the expression of *FLO2* in response to HT was different between cultivars, which indicated that *FLO2* may be involved in heat tolerance during the grain filling stage (She et al., 2010). *FLO11* encodes the heat shock protein OsHsp70-2, whose expression was sensitive to HT. More chalky grains were generated in the *flo11* mutant than in the WT when the rice was grown at 28°C but not at 24°C, indicating that *FLO11* may function under elevated temperature at the milky stage (Tabassum et al., 2020). HSPs (heat shock proteins) are molecular chaperones that delay irreversible aggregation of denatured proteins under HT condition or other stress. The expression of *HSP* is regulated by HSF (heat shock transcription factors) whose activity was affected by Ca^2+^ sensor calmodulin (CaM) in plants (Wu et al., 2012; Bourgine and Guihur, 2021). Transcripts of several *HSP* genes could be induced by HT (Sarkar et al., 2009), which suggested that many HSPs, HSFs and CaMs in the Ca^2+^–dependent heat shock signaling pathway might be essential for acquired thermotolerance of rice quality.

Dynamic analysis of gene expression in the rice endosperm revealed that *α-amylases*, such as *Amy1A*, *Amy3A*, and *Amy3E*, were greatly induced by HT. Knocking down these *α-amylases* significantly improved rice appearance quality under HT (Figure 3B; Hakata et al., 2012), which suggested that *α-amylases* might play key roles in the formation of grain quality under HT. The induced expression of starch-hydrolyzing *α-amylases* implied that a high speed of starch degradation might be another important cause of the increased grain chalkiness under HT conditions. The transcription factor OsbZIP58 might be an essential regulator of *α-amylases*. Knocking out *OsbZIP58*, the expression of *Amy1A*, *Amy3A*, *Amy3E*, and *Amy1C* could be increased, and *osbzip58* mutants produced more chalky grains than WT at HT (Xu et al., 2020). It seems that *OsbZIP58* is an effective suppressor of *α-amylases* in rice endosperm. It might be beneficial to increase the expression of *OsbZIP58* under HT to improve the appearance quality of rice.

## Conclusion and Perspective

Rice quality is a complex trait that covers biochemical, cooking, eating, nutritional, and sensory properties. Starch structure and composition largely determine rice quality, as starch is the major storage material in endosperm. Increasing consumer preference and market demand requires fine control of starch, especially the AC. Although several structural genes, chemical pathways, and regulatory networks involved in starch biosynthesis have been identified in the past few decades, the molecular mechanisms of fine control of starch metabolism remain unclear, which limits the possibility of breeding more diverse and better quality rice. It is still a major challenge for us to establish a precise genetic basis and regulatory network for grain quality, and many open questions remain to be addressed in the future. First, the AC has a decisive effect in grain quality control, and *Wx* is the determinant gene. Although the *Wx* gene has been verified to be finely regulated at multiple levels and an increasing number of essential factors have been isolated (Table 2), most regulatory mechanisms are missing. The lack of fine resolution about crystal structure and post-translational regulation of the GBSSI protein greatly limits our understanding of how to modify its activity. Moreover, in recent decades, many QTLs responsible for rice AC and many novel genes responsible for seed development have been reported. Characterization of these QTLs and genes will be immensely beneficial for clarifying the molecular mechanism of starch biosynthesis and AC control. Second, although many starch synthesis enzymes have been identified and a starch synthesis model has been established for a long time, recent research progress has provided new insights into the function of several starch synthesis enzymes, such as SSs and SBEs. Therefore, more attention should be focused on the novel functions of these starch synthesis enzymes and the physical and genetic interactions between them, which could make the model of starch synthesis more accurate. Finally, grain filling is greatly influenced by HT. The expression pattern and protein activity of many starch synthesis enzymes could be greatly altered under HT. However, only a few QTLs/genes, such as *qHACs* and *OsMADS7*, were recognized as regulatory genes involved in starch metabolism under HT. More genes and regulatory networks are expected to be explored, which will greatly contribute to breeding heat-stable rice varieties with high quality in the future.

**Table 2:** Essential regulators for seed development and amylose content of rice.

Gene name	Gene name synonyms	Locus	Amylose content of mutant/RNAi	Description of gene function	Reference
*OsBP5*	*OsPIL12, OsbHLH103*	LOC_Os03g43810	Decreased	AP2/EREBP transcription factor, interact with OsEBP89 and synergistically regulate the transcription of *Wx*	Zhu et al., 2003
*OsbZIP58*	*RISBZ1*	LOC_Os07g08420	Decreased	Basic leucine zipper transcriptional activator, regulation of *Wx* expression	Wang et al., 2013; Xu et al., 2020
*Du1*	–	LOC_Os10g35550	Decreased	A member of pre-mRNA processing (Prp1) family; Splicing of *Wx* gene	Zeng et al., 2007
*Du3*	*OsCBP20*	LOC_Os02g39890	Decreased	Similar to Nuclear cap binding protein subunit 2; Splicing of *Wx* gene	Isshiki et al., 2008
*OsGBP*	–	LOC_Os02g04330	Decreased	CBM48 domain-containing protein; Mediation of the localization of GBSSs to starch granules	Wang et al., 2020
*FLO6*	–	LOC_Os03g48170	Decreased	CBM48 domain-containing protein; Mediation of the localization of ISA to starch granules	Peng et al., 2014
*OsNF-YB1*	*OsLEC1*	LOC_Os02g49410	Decreased	Nuclear factor Y (NF-Y) transcription factor; regulation of *Wx* expression	Xu et al., 2016; Bello et al., 2019; Xiong et al., 2019
*NF-YC12*		LOC_Os10g11580	Decreased	NF-Y transcription factor subunit C; interact with OsNF-B1and synergistically regulate the transcription of *Wx* and *FLO6*	Bello et al., 2019; Xiong et al., 2019
*bHLH144*	*OsZOU-1*	LOC_Os04g35010	Decreased	Basic helix–loop–helix transcription factor; interact with OsNF-B1and NF-YC12 and synergistically regulate the transcription of *Wx*	Bello et al., 2019
*FLO10*	–	LOC_Os03g07220	Unknown	P-type pentatricopeptide repeat (PPR) protein; Splicing of the mitochondrial gene *NAD1*	Wu et al., 2019
*FLO14*	*OsNPPR3*	LOC_Os03g51840	Not changed	P-type pentatricopeptide repeat (PPR) protein; Splicing of mitochondrial genome-encoded genes	Xue et al., 2019
*FLO18*	–	LOC_Os07g48850	Decreased	P-type pentatricopeptide repeat (PPR) protein; Splicing of the mitochondrial gene NAD5	Yu et al., 2020a
*FLO13*	*OsNDUFA9*	LOC_Os02g57180	Unknown	Mitochondrial complex I subunit	Hu et al., 2018
*FLO16*	*OsCMDH*	LOC_Os10g33800	Decreased	NAD-dependent cytosolic malate dehydrogenase (CMDH); Involved in redox homeostasis	Teng et al., 2019
*FLO2*	–	LOC_Os04g55230	Decreased	Tetratricopeptide repeat (TPR) domain containing protein; Involved in heat tolerance	She et al., 2010
*FLO4*	*OsPPDKB*	LOC_Os05g33570	Decreased	Pyruvate orthophosphate dikinase	Kang et al., 2005; Zhang et al., 2018b
*FLO11*	*OsHsp70*	LOC_Os12g14070	Not changed	Plastid heat shock protein 70; Involved in heat tolerance	Zhu et al., 2018; Tabassum et al., 2020
*FLO15*	*OsGLYI7*	LOC_Os05g14194	Decreased	Plastidic glyoxalase I	You et al., 2019
*OsFIE1*	–	LOC_Os08g04290	Not changed	Core component of the PRC2; Involved in heat tolerance	Huang et al., 2016; Cheng et al., 2020; Dhatt et al., 2021
*OsFIE2*	–	LOC_Os08g04270	Unknown	Core component of the PRC2	Nallamilli et al., 2013; Li et al., 2014; Cheng et al., 2020
*OsMADS1*	*LHS1, LGY3*	LOC_Os03g11614	Unknown	MADS-box transcription factor; negatively regulated by PRC2	Conrad et al., 2014; Liu et al., 2018; Yu et al., 2018
*OsMADS6*	*MFO1*	LOC_Os02g45770	Decreased	MADS-box transcription factor; negatively regulated by PRC2	Conrad et al., 2014; Yu et al., 2020b
*OsMADS29*	–	LOC_Os02g07430	Decreased	MADS box transcription factor; Regulator of early seed development	Yin and Xue, 2012; Nayar et al., 2013
*OsMADS34*	*PAP2*	LOC_Os03g54170	Decreased	MADS-box transcription factor; negatively regulated by PRC2	Ren et al., 2016
*OsMADS78*	–	LOC_Os09g02830	Unknown	MADS-box transcription factor; interact with OsMADS79, OsMADS87, and OsMADS89; negatively regulated by PRC2	Folsom et al., 2014; Paul et al., 2020
*OsMADS87*	–	LOC_Os03g38610	Unknown	MADS-box transcription factor; interact with and OsMADS89; negatively regulated by PRC2; Involved in heat tolerance	Folsom et al., 2014; Chen et al., 2016; Paul et al., 2020
*OsMADS7*	–	LOC_Os08g41950	More stable at high temperature	MADS-box transcription factor; Involved in heat tolerance	Zhang et al., 2018a

## Author Contributions

HuZ and YZ designed the manuscript. All authors listed have made a substantial, direct and intellectual contribution to the work and approved it for publication.

## Funding

This work was supported by the National Key Research and Development Program of China (2016YFD0100902) and the National Natural Science Foundation of China (31401031).

## Conflict of Interest

The authors declare that the research was conducted in the absence of any commercial or financial relationships that could be construed as a potential conflict of interest.

## Publisher’s Note

All claims expressed in this article are solely those of the authors and do not necessarily represent those of their affiliated organizations, or those of the publisher, the editors and the reviewers. Any product that may be evaluated in this article, or claim that may be made by its manufacturer, is not guaranteed or endorsed by the publisher.
